# Towards spatially resolved magnetic small-angle scattering studies by polarized and polarization-analyzed neutron dark-field contrast imaging

**DOI:** 10.1038/s41598-021-87335-3

**Published:** 2021-04-13

**Authors:** Jacopo Valsecchi, Youngju Kim, Seung Wook Lee, Kotaro Saito, Christian Grünzweig, Markus Strobl

**Affiliations:** 1 Laboratory for Neutron Scattering and Imaging, Paul Scherrer Institut, Villigen, Switzerland; 2 University of Geneva, Geneva, Switzerland; 3 School of Mechanical Engineering, Pusan National University, Busan, South Korea

**Keywords:** Applied physics, Condensed-matter physics, Magnetic properties and materials, Physics, Techniques and instrumentation, Imaging techniques

## Abstract

In the past decade neutron dark-field contrast imaging has developed from a qualitative tool depicting microstructural inhomogeneities in bulk samples on a macroscopic scale of tens to hundreds of micrometers to a quantitative spatial resolved small-angle scattering instrument. While the direct macroscopic image resolution around tens of micrometers remains untouched microscopic structures have become assessable quantitatively from the nanometer to the micrometer range. Although it was found that magnetic structures provide remarkable contrast we could only recently introduce polarized neutron grating interferometric imaging. Here we present a polarized and polarization analyzed dark-field contrast method for spatially resolved small-angle scattering studies of magnetic microstructures. It is demonstrated how a polarization analyzer added to a polarized neutron grating interferometer does not disturb the interferometric measurements but allows to separate and measure spin-flip and non-spin-flip small-angle scattering and thus also the potential for a distinction of nuclear and different magnetic contributions in the analyzed small-angle scattering.

## Introduction

The technique of grating interferometry has been introduced to neutron imaging in 2006 as a tool for phase contrast imaging and it has gained substantial impact with the establishment of the neutron dark-field contrast modality, enabling structural studies beyond direct spatial image resolution^1–5^. Dark-field contrast imaging (DFI) provides contrast based on local small-angle scattering (SAS) in bulk samples and thus enabled the visualization of local microstructural inhomogeneities^6–8^. Besides microstructures like pore distributions and precipitation in bulk condensed matter, in particular magnetic structures such as in particular domain walls were found to provide significant contrast^7,9–26^. The latter enabled outstanding studies of magnetic materials and seminal access to 3D domain structures in the bulk of magnetic materials^27–31^.

However, it was significantly later, about half a decade ago, that the quantitative characterization of microstructures based on the probed small-angle neutron scattering in dark-field contrast imaging was unlocked^32^. A scan of the probed structural correlation length $(\xi )$ is achieved through variation of either the applied wavelength $(\lambda )$ of the neutron beam or the distance of the sample to the analyzer grating $(\mathrm { L_s})$, respectively the detector, (in some cases even the period of the modulation^3,32–35^), according to the relation^36^:

1 $$\begin{aligned} \xi = \frac{\lambda \mathrm { L_s}}{p}, \end{aligned}$$

where *p* is the period of the intensity modulation exploited for the small-angle scattering resolution. The basic principle of interferometric dark-field contrast neutron imaging is the introduction of a microscopic transversal intensity modulation in a pinhole collimated beam with a relatively large cross section of several square centimeters as typically used for neutron imaging, and the observation of local visibility loss of the modulation as a consequence of neutron scattering to small angles of the order of $\theta =\frac {p}{\mathrm { L_s}}$^36^. Note that the origin of the beam modulation does not need to be interferometric, like in the conventionally applied Talbot-Lau (TL), and that modulation methods other than TL interferometers and gratings in general have been demonstrated for quantitative neutron dark-field contrast imaging as well^1,24,32–34,37–42^.

**Figure 1:**
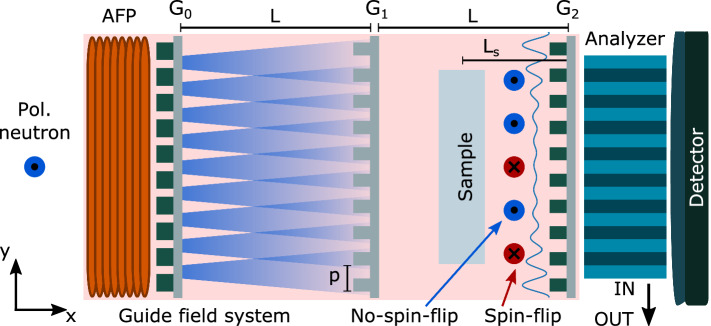
Schematic of the symmetric Talbot–Lau polarized neutron grating interferometer setup including the optional polarization analyzer. The setup consists of an adiabatic fast passage spin flipper (AFP), a source grating $(\mathrm {G_0})$, a phase grating $(\mathrm {G_1})$, an analyzer grating $(\mathrm {G_2}$, a polarization analyzer, a neutron detector and a guide field system (shaded pink). The spin analyzer can be moved out of the beam to the side, i.e. along the y-direction (IN and OUT). The probed polarization direction is along the z-axis. The magnetic sample is placed between $\mathrm {G_1}$ and $\mathrm {G_2}$ at a distance $\mathrm { L_s}$ from the latter one and moved along the x-axis direction to perform a correlation length scan. The inter-grating distance in a symmetric setup are equal to L and all periods are same (*p*). In the sketch the spin-flip and non-spin-flip small-angle scattering events are highlighted symbolically.

The most conventional TL setups consist typically of three gratings referred to as source grating $(\mathrm {G_0})$, phase grating $(\mathrm {G_1})$ and analyzer grating $(\mathrm {G_2})$. The source grating $\mathrm {G_0}$ has a period sufficient to create a transversal coherence at the phase grating $\mathrm {G_1}$ to obtain a cosine interference pattern at the analyzer grating $\mathrm {G_2}$. For dark-field contrast imaging the local visibility (*V*), defined as:

2 $$\begin{aligned} V = \frac{I_{max} - I_{min}}{I_{max} + I_{min}}, \end{aligned}$$

where $I_{max}$ and $I_{min}$ are the transmitted intensities measured at the maximum and minimum points of the beam modulation, is measured. Typically, the open beam visibility range is 15 % ~75 %  and the initial visibility $V_0$ depends not only on the attenuation pattern of $\mathrm {G_0}$ and $\mathrm {G_2}$, but alse the phase shift of $\mathrm {G_1}$ (typically $\frac {\pi }{2}$ or $\pi$ for a certain design wavelength) has to be optimized. In general the geometry is optimized such that the diffraction patterns from beams originating from different source slits add constructively at $\mathrm {G_2}$ placed at a fractional Talbot distance $d_T$ downstream of $\mathrm {G_1}$, as shown in Fig. 1.

The need for covering a useful range of correlation lengths despite the strict wavelength dependence of the interference (due to wavelength dependent phase shift at $\mathrm {G_1}$) has lead to a number of advanced realizations of the original TL interferometer exploiting e.g. higher order Talbot distances $d_{Tn} = n d_{T}$, where $n = 1,3,5$ or moving towards symmetric setups with larger grating periods^1,24,38,43^. Thus, correlation length ranges from 100 nm to about $10 \upmu \hbox {m}$ have become accessible for quantitative dark-field contrast neutron imaging studies in two and three dimensions with TL grating interferometers^39,43–45^.

It has been shown, that upon scanning the parameter $\xi$ through variation of, in general, the wavelength $\lambda$ and the sample to $\mathrm {G_2}$ distance $\mathrm { L_s}$ (compare Eq. 1), the projected correlation function $G(\xi )$ of the scattering structure is measured as^36,46^:

3 $$\begin{aligned} DFI (\xi ) = \frac{V_s(\xi )}{V_0(\xi )} = e^{\Sigma t (G(\xi ) - 1)}, \end{aligned}$$

where $\Sigma$ is the macroscopic scattering cross-section, *t* the sample thickness $V_s$ and $V_0$ the visibility with and without the sample, respectively. Despite the significant success in qualitative dark-field contrast imaging of magnetic structures, quantitative studies of magnetic microstructures do not appear to have been reported so far. It can be assumed that the majority of reported dark-field contrast measurements of magnetic structures, in particular of magnetic domain walls, are originating in fact in oppositely oriented differential phase signals of spin-up $\big | \uparrow \big \rangle$ and spin-down $\big | \downarrow \big \rangle$ spin states of initially unpolarized incident neutrons.

Imaging with polarized neutrons has significantly progressed since its first implementation and is has been demonstrated only recently that TL interferometric imaging with polarized neutrons enables the assessment of strong magnetic fields and gradients which are not amenable to polarized neutron imaging otherwise^47–56^.

Here we introduce a setup where the polarized neutron grating interferometer is extended by polarization analyses in order to probe local polarized small-angle scattering and distinguish nuclear and magnetic structure contributions. The polarization analyzer, a solid state polarizing bender is used, is placed between the analyzer grating $\mathrm {G_2}$ and the detector. Here, the modulation has already been detected by the analyzer grating and therefore no negative impact on the modulation measurement providing dark-field and differential phase contrast is expected. The only impact is thus a somewhat extended detector distance and the related effect on spatial image resolution. However, the utilized polarization analyzer is rather compact with a length of 40 mm along the beam, and the effect on image resolution is therefore negligible against the background of commonly applied spatial resolution requirements in quantitative neutron dark-field imaging to date. This is also related to the general need for variation of $\mathrm {L_s}$ for scanning the correlation length parameter probed. In our application a distance scan range of 200 mm is to be related to the addition of 50 mm for the polarization analyzer with an impact on the spatial resolution of about 15%. We apply scans of the correlation length through scanning the sample to $\mathrm {G_2}$ distance according to values of $\mathrm {L_s}$ being 13, 26, 51, 102 and 202 mm. This corresponds to correlation length values $\xi$ of 98, 195, 387, 768 and 1524 nm.

**Figure 2:**
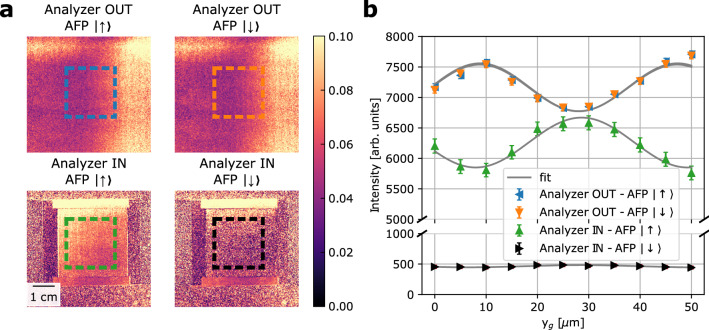
Modulation visibility for polarization-analyzed DFI. **(a)** Visibility maps for the symmetric Talbot-Lau polarized neutron grating interferometer setup in four configurations: without the polarization analyzer in place and spin-up $\big | \uparrow \big \rangle$ (top left), without the polarization analyzer in place and spin-down $\big | \downarrow \big \rangle$ (top right), with the polarization analyzer in place and spin-up $\big | \uparrow \big \rangle$ (bottom left) and with the polarization analyzer in place and spin-down $\big | \downarrow \big \rangle$ (bottom rigth). **(b)** Intensity modulation for the 4 different color coded configurations reported in **(a)** and the corresponding fitted sinusoidal curves. The color coded boxes in **(a)** delimit the fitted area for the intensity modulation fitting.

## Results

The first relevant parameter to asses is the impact of the polarization analyzer and the spin flipper for the incident polarization on the visibility of the beam modulation, which can be exploited. Measurements were performed without sample without spin flip, with spin flip and with the analyzer in the beam. A combination of spin flip and analyzer can naturally not be probed without sample, as the transmission through the analyzer, which under optimal conditions would be zero in such configuration, would deplete the detected beam. This combination, however, allows a measurement of the polarization (P), which can be given with 87.5 %. The results for the modulation are depicted in Fig. 2a, b and demonstrate that no significant impact on the modulation and in particular the visibility is evident by adding the spin-flip and especially the polarization analyses. As it becomes evident, that the spin-flip does not influence the recorded interference pattern, the addition of the analyzer lowers the average intensity by about a fraction of 10% as can be expected due to overall polarization quality and transmission of the analyzer. The achieved visibility remains nevertheless unaltered and remains at about 5% for the indicated central beam area in Fig. 2a, b. This value for the visibility is somewhat lower than for conventional setups, but it has to be noted that measurements here were performed with a white beam, i.e. utilizing the full spectrum available in the instrument^57^. This lowers the contrast but increases equally the count rate. It has been calculated that the effective wavelength thus is 3.77 Å in this setup^55^. The effective wavelength resolution of the measurements can be assumed to be about $\frac {\Delta \lambda }{\lambda } = 30 \%$, which coincides about with the step-width of our $\xi$-scans in the subsequent sample measurements, and thus the $\xi$ resolution in these. Note that the obvious phase shift of the modulation by about half a period that is observed is not a systematic effect, but could be confirmed to be due to an instability induced by the insertion of the analyzer, most likely corresponding to a shift of a few $\upmu \hbox {m}$ of the analyzer grating.

Given these results the setup seems to be capable to measure $G(\xi )$, including the spatial correlations of magnetic structures equivalent to polarized small-angle neutron scattering (SANS). In polarized SANS the sample magnetism is considered to consist of a continuous magnetization vector field $\mathbf {M}(r)$ which represents the magnetization state of the sample at each position *r* inside the material^58^. Magnetic SANS can thus be assumed an implication of nanoscale variations in the magnitude or orientation of the magnetization, or both, representing a magnetic structure. Here, we are using uniaxial polarization analysis which enables to measure four different scattering quantities which are related to the two probed neutron spin states spin-up $\big | \uparrow \big \rangle$ and spin-down $\big | \downarrow \big \rangle$. The orientation of the incident polarization and polarization analyses defines the quantization axis for the scattered polarization. An externally applied magnetic field $\mathbf {H}$ at the sample position, as well as the guide field of the instrument, is aligned correspondingly and along the easy axis of the magnetization. The scattered neutron can either conserve the initial polarization direction in the scattering process described by the cross sections $\frac {d\Sigma ^{++}}{d\Omega }$ and $\frac {d\Sigma ^{--}}{d\Omega }$ for spin-up $\big | \uparrow \big \rangle$ and spin-down $\big | \downarrow \big \rangle$ incident polarizations, respectively, or it undergoes spin-flip scattering when undergoing a spin-reversing event due to the magnetic interaction with the sample according to $\frac {d\Sigma ^{+-}}{d\Omega }$ and $\frac {d\Sigma ^{-+}}{d\Omega }$. While in SANSPOL, referring to SANS with polarized neutrons, but no polarization analysis capability, only half-polarized cross sections are probed, the addition of polarization analysis enables all individual cross sections to be assessed separately. The corresponding expressions for the cross sections are denoted as the POLARIS equations and can be written as^58^:

4 $$\begin{aligned} \frac{d\Sigma ^{+}}{d\Omega } = \frac{d\Sigma ^{++}}{d\Omega } + \frac{d\Sigma ^{+-}}{d\Omega }, \quad \frac{d\Sigma ^{-}}{d\Omega } = \frac{d\Sigma ^{--}}{d\Omega } + \frac{d\Sigma ^{-+}}{d\Omega }. \end{aligned}$$

**Figure 3:**
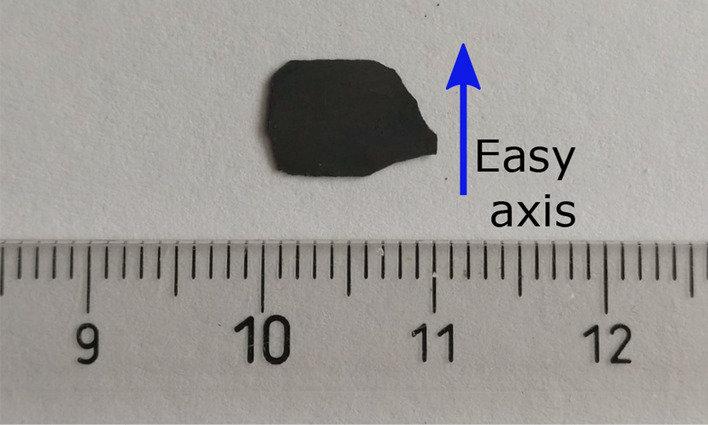
Photographic image of the NdFeB sintered magnetic sheet. The sintered magnet is characterized by big and isotropic grains of few micrometer in size. The sample is 0.15 mm thick and coated with 0.5 nm tantalum. The blue arrow indicates the easy axis of the magnetization. The scale in the background is expressed in (cm).

In our setup the incident polarization can be approximated by $\mathbf {P} = \mathbf {e}_{\mathrm {z}}$ where $\mathbf {e}_{\mathrm {z}}$ is the unit vector in the vertical direction. The incident wave vector $|{\mathbf {k}}|= k_{\mathrm {x}}$ in x-direction together with the horizontal transversal direction y define the scattering plane (x-y) where $|{\mathbf {q}}|= q_{\mathrm {y}}$ is the scattering vector providing dark-field contrast. According to the magnetic interaction vector, underlining the dipolar origin of magnetic neutron scattering, only components of the magnetization ${\mathbf {M}}(r)$ perpendicular to the scattering vector $\mathbf {q}$ contribute to magnetic scattering^59^. Thus, only $\mathbf {M}_{\mathrm {x}}$ and $\mathbf {M}_{\mathrm {z}}$ are probed in our setup. In order to investigate the principle potential of polarized SANS^58^ investigations with our polarization analyzed grating interferometer in dark-field contrast imaging we measured an initially demagnetized NdFeB ferromagnetic sample, shown in Fig. 3, with polarized neutrons and polarization analyses. This included the optional application of a magnetic field $\mathbf {H}_{\mathrm {z}}$ of 0.7 T at the sample position.

**Figure 4:**
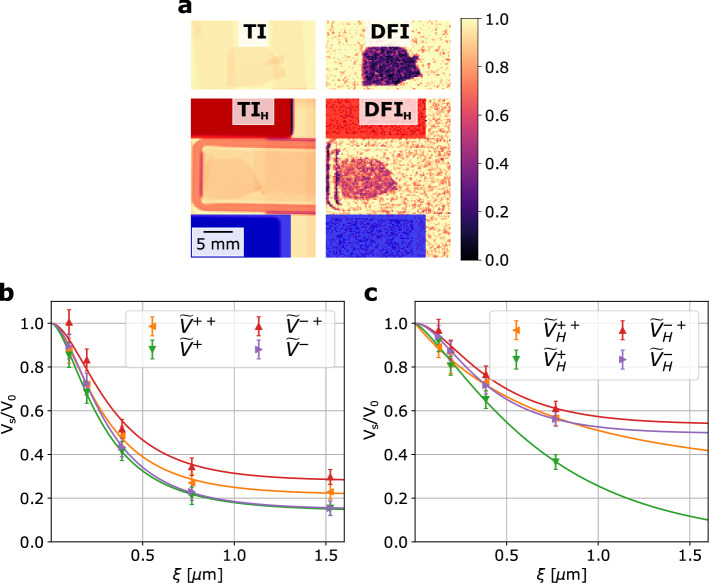
Measured polarized DFI of the sintered NdFeB magnet. **(a)** TI and DFI images of the NdFeB with and without the external magnetic field at $\xi \simeq 0.7 \upmu \hbox {m}$ and without analyzer in place. The North and the South poles of the applied field are depicted in red and blue, respectively. **(b)** DFI experimental values and the fitted $\frac {V_s(\xi )}{V_0}$ curves based on model of correlation functions $G(\xi )$ for the four configurations without the external magnetic field corresponding to the $V^{++}$, $V^{-+}$, $V^{+}$ and $V^{-}$. **(c)** DFI experimental values and the fitted DFI curves with the extracted correlation functions $G(\xi )$ for the four configurations with the external magnetic field corresponding to the $V^{++}_{H}$, $V^{-+}_{H}$, $V^{+}_{H}$ and $V^{-}_{H}$. The modeled $G(\xi )$ functions were based on the description of random two phase media. The magnetization easy axis of the sample is along the short side of the sample and along the applied external magnetic field.

Our polarization analyzer was aligned to probe the spin-up state $\big | \uparrow \big \rangle$ $(\mathbf {P}_{\mathrm {z}})$, but it could be removed from the beam, in order to probe the half-polarized scattering cross sections according to Eq. (4). The sum of both provides the unpolarized scattering. Combination with the polarized cross sections probed with the polarization analyzer, i.e. $\frac {d\Sigma ^{++}}{d\Omega }$ and $\frac {d\Sigma ^{-+}}{d\Omega }$, thus in principle enable to recover all components as $\frac {d\Sigma ^{+}}{d\Omega } - \frac {d\Sigma ^{++}}{d\Omega } = \frac {d\Sigma ^{+-}}{d\Omega }$ and $\frac {d\Sigma ^{-}}{d\Omega } - \frac {d\Sigma ^{-+}}{d\Omega } = \frac {d\Sigma ^{--}}{d\Omega }$, according to Eq. (4). The measurements are summarized in Fig. 4a–c.

Correspondingly, we measure $V^{+}$, $V^{-}$, $V^{++}$ and $V^{-+}$, where $V^{\pm } = \frac {V^{\pm }_s}{V^{\pm }_0}$, the visibility of the sample $V^{\pm }_s$ normalized by the corresponding open beam visibility without sample $V_0^{\pm }$. The ± superscripts refer to the incident and analyzed polarization direction in full analogy to the cross sections before. In case of $V^{-+}$ no corresponding $V_0$ can, however, be measured (compare the case of (i) Analyzer IN - AFP $\big | \downarrow \big \rangle$, in Fig. 2a). Therefore, we define $V^{-+} = \frac {V^{-+}_s}{V^{++}_0}$ with the apparently justified assumption that $V^{+}_0 = V^{++}_0 = V^{-+}_0$. Considering the magnetic interaction vector and our scattering geometry the half-polarized cross sections include contributions of nuclear scattering, and interactions with $\mathbf {M}_{\mathrm {x}}$ and $\mathbf {M}_{\mathrm {z}}$ as well as a cross term of nuclear structure and $\mathbf {M}_{\mathrm {z}}$ scattering differently impacting $\Sigma ^{+}$ and $\Sigma ^{-}$, respectively. The polarized neutron scattering term $\Sigma ^{++}$ consists similarly of the nuclear and $\mathbf {M}_{\mathrm {z}}$ terms, including the cross term, but without contributions of $\mathbf {M}_{\mathrm {x}}$. The $\Sigma ^{-+}$ related data, on the other hand, probes solely the $\mathbf {M}_{\mathrm {x}}$ related contribution.

While we observe significant scattering in the probed length scale regime, only minor differences in the measured terms are found before we apply the external magnetic field, as shown in Fig. 4b,c. When applying the external field $\mathbf {H}_{\mathrm {z}}$ (about 0.7 T throughout the sample region, as shown in Fig. 2a) the scattering is reduced for all components, but the least in case of $\Sigma ^{+}$ which differs from $\Sigma ^{-}$ only with respect to the cross term of $\mathbf {M}_{\mathrm {z}}$ with the structural contribution. While in the case without magnetic field the correlation functions overall appeared to nearly saturate within the probed range, hinting for maximum correlation lengths of about the end of our scale $(1.5 \upmu \hbox {m})$, larger correlation lengths appear to have been induced parallel to $\mathbf {q}_{\mathrm {y}}$ with the applied field indicating growth of $\mathbf {M}_{\mathrm {z}}$ domains.

**Figure 5:**
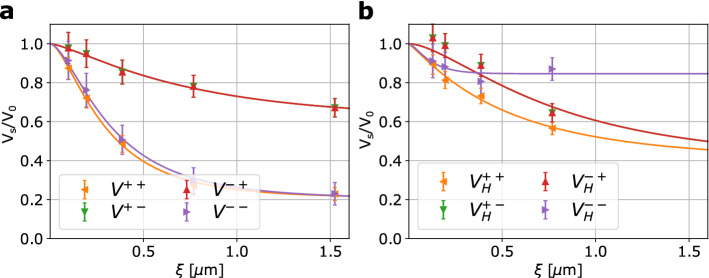
**(a)** Retrieved $V^{+-}$ and $V^{--}$ from the measured $V^{+}$, $V^{-}$ and $V^{++}$, without the external magnetic field. **(b)** Retrieved $V^{+-}_{H}$ and $V^{--}_{H}$ from the measured $V^{+}_{H}$, $V^{-}_{H}$ and $V^{++}_{H}$, with the applied external magnetic field. The $G(\xi )$ functions were extracted by fitting the model for random two phase media.

In order to better disentangle the measured information, it was attempted to retrieve the four contributions according to the polarized SANS cross sections of non-spin-flip and spin-flip scattering. While in SANS subtractions of the measured cross sections are appropriate, here we measure the cross section in the exponent, thus we use division instead. Accordingly, we retrieve, in addition to the directly measured components of $\Sigma ^{++}$ and $\Sigma ^{-+}$ the DFI signal corresponding to $\Sigma ^{+-}$ and $\Sigma ^{--}$ by $V^{+-} = \frac {V^+}{V^{++}}$ and $V^{--} = \frac {V^-}{V^{-+}}$. The thus retrieved curves are depicted in Fig. 5a,b, without and with external field $\mathbf {H}_{\mathrm {z}}$, respectively.

In the above approximation of considering $\mathbf {q}_{\mathrm {y}}$ only, we can, in principle, also assume that $V^{-+} \approx V^{+-}$ because both spin-flip components equally probe, under such assumption, only $\mathbf {M}_{\mathrm {x}}$. However, our data has the biggest difference for particularly these components. $V^{-+}$, on the other hand, carries a bias that corresponds to an overestimation of the measured scattering, because, while in DFI the scattering is measured as a component on top of the transmitted, thus unscattered beam, for $V^{-+}$ this unscattered component, that constructively contributes to the visibility is filtered out by the polarization analyzer.

Therefore, it is attempted to reproduce the four DFI curves based on the above assumption of correspondence of $V^{+-}$ and $V^{-+}$ only from $V^{+}$, $V^{++}$ and $V^{-}$. The corresponding curves are presented in Fig. 5, while a direct comparison of the measured $V^{-+}$ and the reconstructed $V^{+-}$ is shown in the Supplemental Material.

It is in this case observed, that $V^{++}$ and $V^{--}$ are equal in the field free case. That implies that the scattering is indifferent to the orientation of the incoming spin direction, and thus the magnetization, i.e. domain orientation is completely random also with respect to the nuclear scattering due to e.g. porosity of the sample. Upon application of the field $\mathbf {H}_{\mathrm {z}}$, however, the situation changes and different correlations of the nuclear structure with respect to the magnetization structure cause a deviation of $V^{++}$ and $V^{--}$ from each other. Notably the $V^{--}$ curve appears to saturate already at correlation lengths of about 200 nm. With the growth of the domains oriented parallel to the applied field and the opposite effect on the antiparallel orientation, the total scattering decreases. Turning to the spin-flip component $V^{+-}$ we find that the application of the magnetic field has a relatively small impact on the probed $\mathbf {M}_{\mathrm {x}}$ correlation function, overall mostly within the range of error bars associated. The lack of the last correlation length measured for this sample condition adds uncertainty. It could be speculated, as the sample is not in saturation magnetization, that residual misoriented domains contribute to this signal up to considerably long length scales.

## Discussion

In conclusion, it has been demonstrated that polarization analyses can be added to Talbot-Lau grating interferometers for dark-field contrast imaging. The addition of a polarization analyzer downstream the analyzer grating $\mathrm {G}_2$ does not entail a penalty in visibility or functionality. It has further been shown that a Talbot-Lau grating interferometer operated with polarized neutrons and polarization analyses capability enables to probe not only dark-field contrast from half-polarized SANS, but to also separate and retrieve dark-field contrast for all spin-flip and non-spin flip small-angle neutron scattering components separately. It has, however, also been found that the directly measured spin-flip component of the dark-field contrast carries a bias overestimating the spin-flip scattering due to removal of the transmitted, unscattered part of the beam, which carries the incident polarization. This effect cannot straightforwardly be corrected, or compensated for, in quantitative dark-field contrast imaging studies. A method to circumvent the issue in a first approximation has been applied, based on the assumption, that only scattering in the horizontal plane is probed in the presented set-up. However, this is not strictly the case, because what is referred to as slit-smearing in conventional SANS methods indeed introduces also scattering out of this plane to the recorded signal. This consideration, however, opens a whole different dimension of complexity in the interpretation of results as the ones presented and, thus, must remain for consecutive efforts to enable quantitative polarized SANS with spatial resolution in neutron grating interferometry. So far different approaches have been applied and proposed in DFI to assess anisotropic scattering, which include the rotation of the sample or grating set-up around the beam axis, or 2D gratings respectively^42,60^. It remains to be seen how well suited such approaches are for polarized DFI. Here, however, we have presented seminal results demonstrating the principle potential and instrumental development alongside a discussion of remaining issues that need to be solved in order to enable a full spatially resolved characterization of magnetic structures from polarization analysed dark-field contrast imaging.

Finally, the presented set-up of combined Talbot–Lau interferometry and polarization analyses will enable combined dark-field contrast and polarization contrast imaging for the visualization of magnetic domain walls and characterization of the domain magnetization, respectively, which today requires separate measurements with different instrumentation^11,13,16–18,24,25,31,54^. In such case, however, the polarization analyses might be oriented perpendicular to the grating orientation, in contrast to the here presented geometry. A geometry where the applied external field, the polarization and the probed scattering vector are aligned perpendicular to the beam, on the other hand could be suited to separate nuclear and magnetic scattering components, which could prove highly useful for the investigation of microstructures of magnetic materials^58^. When, on the other hand, applying the field parallel to the beam parallel and perpendicular magnetization components can be discriminated straight forwardly.

## Methods

### Symmetric Talbot–Lau polarized neutron grating interferometer setup with optional polarization analyzer

We employed a symmetric TL grating setup, as shown in Fig. 1, with periods *p* equals to $50 \upmu \hbox {m}$ for all three gratings^38,39^. The setup was installed at the beamline BOA of the continuous spallation neutron source SINQ of the Paul Scherrer Institut (PSI) in Switzerland^61^. The total length of the grating setup $2 \mathrm {L_s}$ was 2.84 m. The beam provided by the BOA instrument is polarized already by a polarizing bender neutron optics in the extraction section in the biological shielding of the source. The polarizing bender, facing a cold source has a cut-off wavelength of 3 Å at the short wavelength side of the spectrum. The detector used was a $200 \upmu \hbox {m}$ LiF/ZnS scintillator screen coupled via a mirror and 100 mm optics with a CCD digital camera [1024 $\times$ 1024 pixels]. The effective pixel size was $230 \upmu \hbox {m}$ on a field of view of 100 *times* 100 mm^2^, characterized with a Siemens star test object^62^. The field of view for the grating measurements was further limited by the gratings to 64 *times* 64 mm^2^ and finally the polarization analyzer to 40 *times* 40 mm^2^. The bender-type analyzer consists of $120 \upmu \hbox {m}$ thick coated Si lamellae^63^.

### Sintered NdFeB magnetic sheet

The probed sample for the polarized and polarization-analyzed $\xi$DFI measurement is a thin NdFeB sintered magnet. The NdFeB magnetic sheet, shown in Fig. 3, has a rectangular shape of $10 \times 7$ mm^2^, it is 0.15 mm thick and it is coated with thin film of tantalum, 0.5 nm, on both sides, deposited by radio frequency sputtering to prevent oxidation. This kind of sitered magnets are characterized by big and isotropic grains of few micrometer in size, typically $2 \sim 5 \upmu \hbox {m}$, and a crystallographic anisotropy^64–66^. The magnetization easy axis of the NdFeB sintered magnet is along the short side of the sample, as depicted in Fig. 3, and along the applied external magnetic field as shown in Fig. 4. The saturation is reached for magnetic field of 5 T, while a typical coercivity field lies between 1 to 2 T when it’s fully magnetized. However, in our case the sample has been thermally demagnetized by warming up the magnetic sheet above the Curie temperature, T$_C \simeq 620$ K, before the measurement while the maximum external magnetic field applied to the sample was roughly H $\simeq 0.7$ T, far below the saturation.

## Supplementary Information

Supplementary Figure S1.
