# Supplementary material for: Towards spatially resolved magnetic small-angle scattering studies by polarized and polarization-analyzed neutron dark-field contrast imaging
Source: Sci Rep. 2021 Apr 13;11:8023. doi: 10.1038/s41598-021-87335-3 (PMC8044191; doi:10.1038/s41598-021-87335-3)
Supplement: Supplementary file 1 — Supplementary Figure S1. [file 41598_2021_87335_MOESM1_ESM.pdf]

Towards spatially resolved magnetic small-angle scattering studies by polarized and polarization-analyzed neutron dark-field contrast imaging

Jacopo Valsecchi[1,2], Youngju Kim[3], Seung Wook Lee[3], Kotaro Saito[1], Christian Grünzweig[1], and Markus Strobl[1,*]

[1]Laboratory for Neutron Scattering and Imaging, Paul Scherrer Institut, Villigen, Switzerland
[2]University of Geneva, Switzerland
[3]School of Mechanical Engineering, Pusan National University, Busan, South Korea
[*]markus.strobl@psi.ch

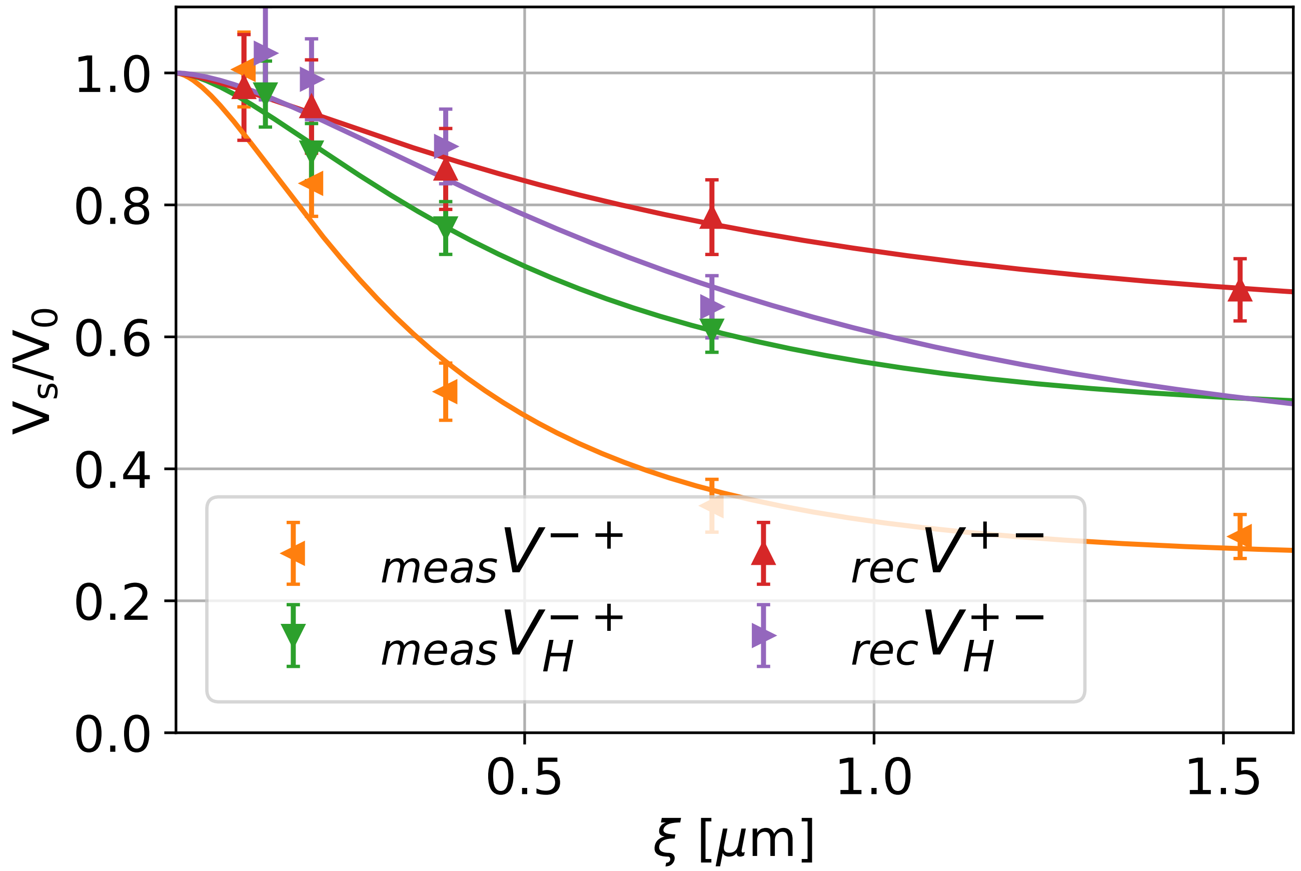

Supplementary Figure 1. Comparison of the measured $_{meas}V^{-+}$ and the retrieved $_{rec}V^{+-}$ polarized DFI of the sintered NdFeB magnet, with and without the applied external magnetic field. The modeled $G(\xi)$ functions based on the description of random two phase media are shown accordingly.
